# Supplementary figures and images for: Insights into DNA substrate selection by APOBEC3G from structural, biochemical, and functional studies
Source: PLoS One. 2018 Mar 29;13(3):e0195048. doi: 10.1371/journal.pone.0195048 (PMC5875850; doi:10.1371/journal.pone.0195048)

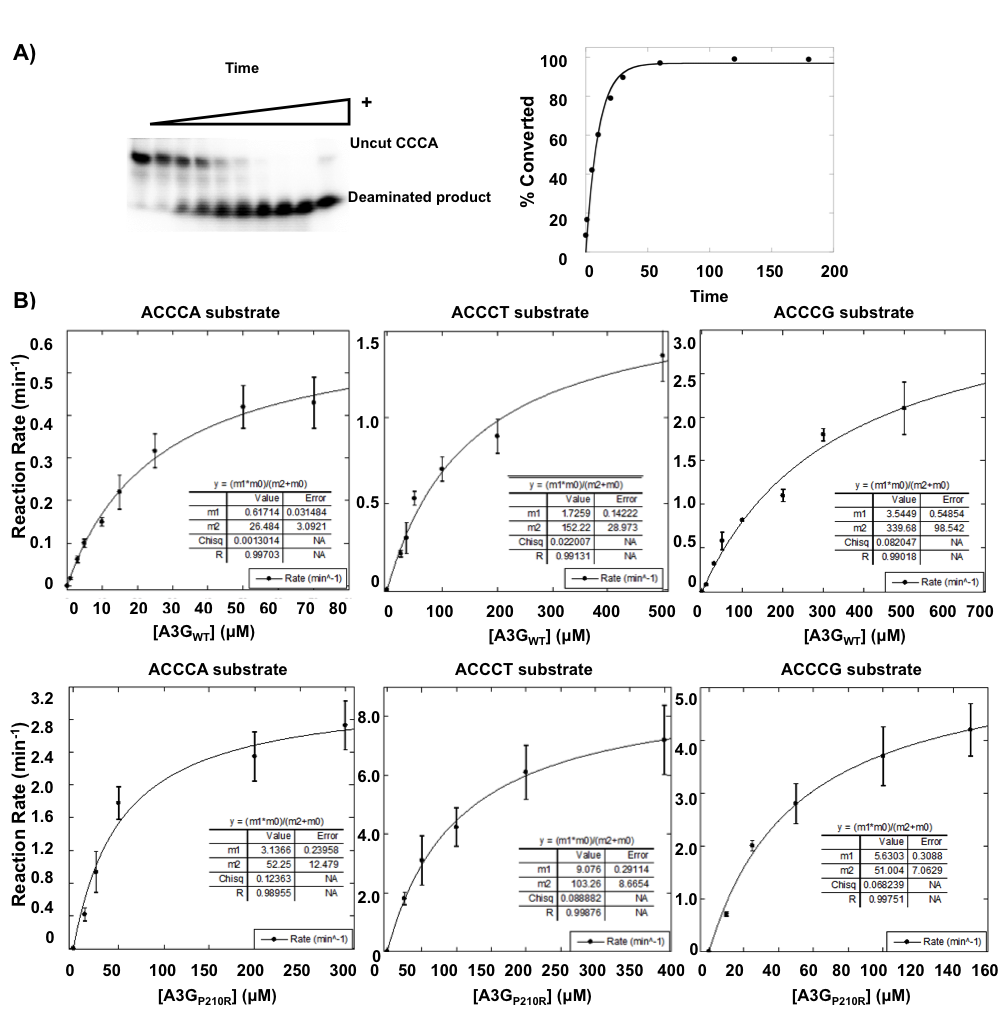

Supplement: S1 Fig — A) Representative kinetic curve to determine the rate of deamination of WT A3GCTD. Shown here is the result for 5μM A3GCTD. B) The kinetics plot results for the WT A3GCTD and P210R A3GCTD reaction on CCCA, CCCG, and CCCG substrates over a range of A3G concentrations. All data were analyzed and summarized in Fig 4D. (TIF) [file pone.0195048.s001.tif]

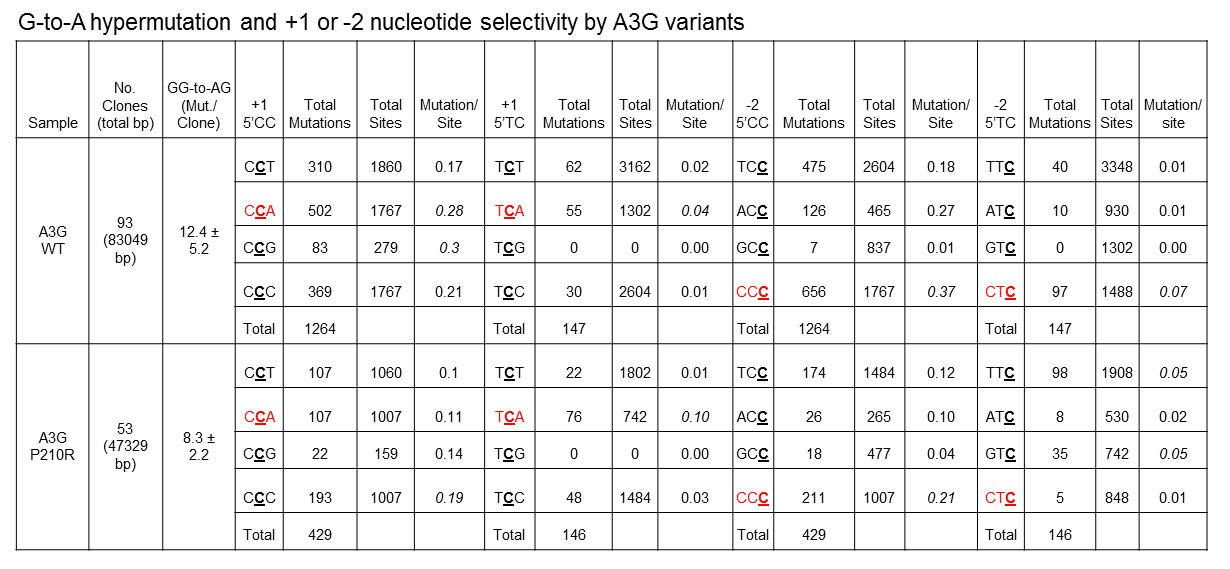

Supplement: S1 Table — Hypermutation is defined as ≥ 2 G-to-A mutations per clone (the no A3G control, on average, had <1 G-to-A mutations per clone). Each clone contained 61 5’CC and 76 5’TC target sites. Mutations/site = total mutations/[sites/clone × no. of clones]. For all conditions, the mutation frequencies for each nucleotide are shown relative to the total mutations/site as determined by the +1 or -2 position nucleotides. Relative preference of nucleotides at the +1 or -2 position in both the 5’-CC and 5’-TC edited sites are plotted for virions produced in the presence of A3G-WT or A3G-P210R. (TIF) [file pone.0195048.s002.tif]
